# Risk factors of recurrence and distant metastasis in primary cutaneous melanoma in Taiwan

**DOI:** 10.1038/s41598-021-00386-4

**Published:** 2021-10-25

**Authors:** Tung-Lin Lee, Yi-Hua Liao, Jau-Yu Liau, Yi-Shuan Sheen

**Affiliations:** 1 Department of Medical Education, National Taiwan University Hospital, Taipei, Taiwan; 2 Department of Dermatology, National Taiwan University Hospital and National Taiwan University College of Medicine, 7 Chung-Shan S. Rd., Taipei, 100 Taiwan; 3 Department of Pathology, National Taiwan University Hospital and National Taiwan University College of Medicine, Taipei, Taiwan

**Keywords:** Cancer, Melanoma, Risk factors

## Abstract

Risk factors of recurrence and distant metastasis of acral lentiginous melanoma (ALM) are of great interest for the high percentage of ALM in cutaneous melanoma in Asian populations. This single-center retrospective cohort including 177 patients with localized melanoma diagnosed from 2004 to 2020 aims to identify adverse predictors in cutaneous melanoma in Taiwan, with a focus on ALM. The relationship between clinicopathological features and outcomes, including incidences of recurrence and distant metastasis in 5 years from diagnosis, was analyzed. This study included 124 patients (70.1%) with ALM and 53 (29.9%) with non-ALM melanoma. Regarding clinicopathological characteristics, ALM patients were diagnosed at an older age and received sentinel lymph node biopsies (SLNBs) more often, while adjacent melanocytic nevi were more prevalent in non-ALM patients. With respect to prognostic implications of clinicopathological features, in ALM, implementation of SLNB was associated with a lower 5-year distant metastasis rate. Thickness of melanoma lesions over 4 mm, ulceration, and neurotropism, were related to both higher 5-year recurrence and distant metastasis rates. With regard to non-ALM patients, diagnoses made at or over 65 years old was linked to a higher 5-year recurrence rate, whereas ulceration was associated with both higher 5-year recurrence and distant metastasis rates. In conclusion, several clinicopathological characteristics have been identified to be associated with poor prognosis of cutaneous melanoma, especially ALM.

## Introduction

In Taiwan, malignant melanoma is a rare entity, accounting for 0.25% of all cases of malignancy. Over 50% of malignant melanoma cases in Taiwan are acral lentiginous melanoma (ALM)^1–3^. This epidemiological observation is consistent with that in other Asian populations^4,5^. ALM patients present with irregularly shaped macules, nodules, or ulcers, which are brownish-to-dark but may also be red with variegations in color. Most commonly, ALM lesions occur on the soles, followed by the palms and subungual areas^6^.

Several lines of evidence have demonstrated that ALM is distinct to other subtypes of melanoma in terms of clinicopathological characteristics as well as risk factors for poor prognosis. Male gender, the presence of amelanotic lesions, and thicker melanoma lesions are known adverse prognostic factors^7–9^. Recent endeavors have been made to identify risk factors for recurrence, distant metastasis, and melanoma-specific survival (MSS) of ALM yet with inconclusive results in literature. In light of the scarcity of related studies, specifically in Asia, we compiled this single-center cohort to investigate the risk of melanoma recurrence, distant metastasis, and shortened MSS in patients with localized ALM at a high risk of metastasis. In this study, clinical and pathological characteristics of ALM and non-ALM patients were compared. On top of that, the associations between clinicopathological variables and endpoints including the incidence of recurrence and distant metastasis in 5 years, as well as 5-year MSS, were analyzed^10–13^.

## Results

### Patient characteristics in ALM and non-ALM cutaneous melanoma

This study included 82 (46.3%) men and 95 (53.7%) women diagnosed with cutaneous melanoma at a mean age of 63.7 years (median: 65 years; range: 20–93 years). One hundred and twenty-four (70.1%) patients were diagnosed with ALM and 53 (29.9%) with non-ALM cutaneous melanoma. In both ALM and non-ALM patients, a slight female preponderance was observed, with no significant difference in male-to-female ratio between the two groups (*p* = 0.8554). Eighty-nine (50.3%) patients were diagnosed with cutaneous melanoma at or older than 65 years of age. Notably, a significant difference existed in the age of diagnosis between ALM and non-ALM patients, with 73 (58.9%) ALM patients and 16 (30.2%) non-ALM patients diagnosed at or over 65 years of age, respectively (*p* = 0.0005). While the primary lesions of ALM arose on acral regions, non-ALM cutaneous melanoma lesions emerged on the trunk (35.9%), head and neck (17.0%), and the extremities excluding the acral regions (47.2%). SLNBs were performed in 105 (84.7%) ALM patients, significantly more frequently than in the 29 (55.8%) non-ALM patients (*p* < 0.0001).

Comparing the pathological characteristics of ALM and non-ALM patients, no discrepancy was found between the two groups regarding the thickness of lesions, ulceration, mitotic rate, lymphovascular invasion, lymphocytic infiltration, tumor regression, neurotropism or desmoplasia. Nevi adjacent to the tumor at diagnosis (40.0% in non-ALM vs. 8.1% in ALM) were more frequently found in non-ALM patients. There was no significant difference in the 8th edition American Joint Committee on Cancer (AJCC) T category or clinical stage at diagnosis between ALM and non-ALM patients. A comparison of clinical and pathological variables between ALM and non-ALM cutaneous melanoma was summarized in Table 1.

**Table 1:** Clinicopathological characteristics of ALM and non-ALM cutaneous melanoma.

Variables	ALM	Non-ALM	Chi-square/Fisher test *P* value
Total, No. (%)	124 (70.1)	53 (29.9)	
**Sex, No. (%)**			
Male	58 (46.8)	24 (45.3)	0.8554
Female	66 (53.2)	29 (54.7)	
**Age, No. (%), years**			
< 65	51 (41.1)	37 (69.8)	0.0005*
≥ 65	73 (58.9)	16 (30.2)	
**SLNB, No. (%)**			
Performed	105 (84.7)	29 (55.8)	< 0.0001*
Not performed	19 (15.3)	23 (44.2)	
**Thickness, No. (%), mm**			
≦ 1	28 (22.6)	15 (28.3)	0.6912
1–2	37 (29.8)	13 (24.5)	
2–4	30 (24.2)	15 (28.3)	
> 4	29 (23.4)	10 (18.9)	
**Ulceration, No. (%)**			
Present	47 (37.9)	14 (26.4)	0.1408
Absent	77 (62.1)	39 (73.6)	
**Mitosis, No. (%), /mm2**			
< 1	36 (40.0)	10 (27.0)	0.3400
1–3	21 (23.3)	12 (32.4)	
≧ 3	33 (36.7)	15 (40.6)	
**Lymphovascular invasion, No. (%)**			
Present	6 (7.2)	1 (3.7)	1.0000
Not identified	77 (92.8)	26 (96.3)	
**Lymphocytic infiltration, No. (%)**			
None	3 (3.9)	1 (3.3)	0.3928
Non-brisk	70 (92.1)	26 (86.7)	
Brisk	3 (3.9)	3 (10.0)	
**Tumor regression, No. (%)**			
Present	11 (15.1)	7 (26.9)	0.2358
Not identified	62 (84.9)	19 (73.1)	
**Neurotropism, No. (%)**			
Present	13 (15.7)	3 (11.1)	0.7565
Not identified	70 (84.3)	24 (88.9)	
**Desmoplasia, No. (%)**			
Present	5 (6.9)	1 (3.7)	1.0000
Not identified	67 (93.1)	26 (96.3)	
**Adjacent melanocytic nevus, No. (%)**			
Present	6 (8.1)	12 (40.0)	< 0.0001*
Not identified	68 (91.9)	18 (60.0)	

*ALM* acral lentiginous melanoma, *SLNB* sentinel lymph node biopsy.

*Statistically significant with Chi-square/Fisher test *P* values < 0.05.

### Clinicopathological characteristics and 5-year recurrence and distant metastasis rates

The relationship between clinicopathological characteristics and the incidence of recurrence and distant metastasis in 5 years in ALM patients are shown in Tables 2 and 3, while that in non-ALM patients are shown in Tables S1 and S2 in Supplementary Material. The 5-year recurrence-free survival (RFS) of ALM and non-ALM patients were 76.6% and 75.5%, respectively. The 5-year distant metastasis-free survival (DMFS) of ALM and non-ALM patients were 80.6% and 83.0%, respectively. The 5-year MSS of ALM and non-ALM patients were 83.9% and 88.7%, respectively. The RFS, DMFS, and MSS of ALM and non-ALM patients stratified by the 8th AJCC T category and clinical stage were summarized in Table 4.

**Table 2:** Clinical risk factors of recurrence and distant metastases in 5 years among ALM patients.

Variables	Total	No recurrence	Recurrence in 5 years	HR (95% CI)	No metastasis	Distant Metastasis in 5 years	HR (95% CI)
Total, No. (%)	124 (100)	95 (76.6)	29 (23.4)		100 (80.7)	24 (19.3)	
**Sex, No. (%)**							
Male	58 (46.4)	46 (48.4)	12 (41.4)	0.94 (0.45–1.97)	49 (49.0)	9 (37.5)	0.83 (0.36–1.91)
Female	67 (53.6)	49 (51.6)	17 (58.6)	Ref	51 (51.0)	15 (62.5)	Ref
**Age, No (%), years**							
< 65	51 (40.8)	40 (42.1)	11 (37.9)	Ref	41 (41.0)	10 (41.7)	Ref
≥ 65	74 (59.2)	55 (57.9)	18 (62.1)	1.33 (0.63–2.83)	59 (59.0)	14 (58.3)	1.06 (0.47–2.39)
**SLNB, No. (%)**							
Performed	106 (84.8)	82 (86.3)	23 (79.3)	0.51 (0.21–1.26)	87 (87.0)	18 (75.0)	0.37 (0.15–0.94)*
Not performed	19 (15.2)	13 (13.7)	6 (20.7)	ref	13 (13.0)	6 (25.0)	ref

*ALM* acral lentiginous melanoma, *SLNB* sentinel lymph node biopsy, *HR* hazard ratio, *CI* confidence interval.

*Statistically significant with 95% CIs not including 1.00.

**Table 3:** Pathological risk factors of recurrence and distant metastases in 5 years among ALM patients.

Variables	Total	No recurrence	Recurrence in 5 years	HR (95% CI)	No metastasis	Distant Metastasis in 5 years	HR (95% CI)
Total, No. (%)	124 (100)	95 (76.6)	29 (23.4)		100 (80.7)	24 (19.3)	
**Thickness, No. (%), mm**							
≤ 1	28 (22.6)	25 (26.3)	3 (10.3)	Ref	25 (25.0)	3 (12.5)	Ref
> 1, ≤ 2	37 (29.8)	34 (35.8)	3 (10.3)	0.76 (0.15–3.75)	34 (34.0)	3 (12.5)	0.75 (0.15–3.71)
> 2, ≤ 4	30 (24.2)	22 (23.2)	8 (27.6)	2.73 (0.73–10.31)	26 (26.0)	4 (16.7)	1.18 (0.26–5.27)
> 4	29 (23.4)	14 (14.7)	15 (51.7)	6.12 (1.77–21.18)*	15 (15.0)	14 (58.3)	5.46 (1.56–19.07)*
**Ulceration, No. (%)**							
Present	47 (37.9)	26 (27.4)	21 (72.4)	5.85 (2.58–13.23)*	29 (29.0)	18 (75.0)	6.45 (2.55–16.32)*
Absent	77 (62.1)	69 (72.6)	8 (27.6)	Ref	71 (71.0)	6 (25.0)	Ref
**Mitosis, No. (%)/mm**^**2**^							
< 1	36 (40.0)	30 (41.7)	6 (33.3)	Ref	31 (40.8)	5 (35.7)	Ref
≥ 1, < 3	21 (23.3)	20 (27.8)	1 (5.6)	0.28 (0.03–2.36)	20 (26.3)	1 (7.1)	0.32 (0.04–2.76)
≥ 3	33 (36.7)	22 (30.5)	11 (61.1)	2.49 (0.92–6.76)	25 (32.9)	8 (57.1)	2.13 (0.70–6.52)
**Lymphovascular invasion, No. (%)**							
Present	6 (7.2)	3 (4.7)	3 (15.8)	2.31 (0.67–7.95)	3 (4.5)	3 (18.8)	3.09 (0.88–10.88)
Not identified	77 (92.7)	61 (95.3)	16 (84.2)	Ref	64 (95.5)	13 (81.3)	Ref
**Lymphocytic infiltration, No. (%)**							
None	3 (3.9)	1 (1.6)	2 (13.3)	ref	2 (3.1)	1 (8.3)	Ref
Non-brisk	70 (92.1)	57 (93.4)	13 (86.7)	0.34 (0.08–1.51)	59 (92.2)	11 (91.7)	0.70 (0.09–5.52)
Brisk	3 (3.9)	3 (4.9)	0 (0.0)	0	3 (4.7)	0 (0.0)	0
**Regression, No. (%)**							
Present	11 (15.1)	8 (13.8)	3 (20.0)	2.07 (0.58–7.42)	9 (14.8)	2 (16.7)	1.73 (0.38–7.92)
Not identified	62 (84.9)	50 (86.2)	12 (80.0)	ref	52 (85.2)	10 (83.3)	Ref
**Neurotropism, No. (%)**							
Present	13 (15.7)	4 (6.2)	9 (50.0)	14.08 (4.65–42.60)*	6 (8.7)	7 (50.0)	12.70 (3.81–42.34)*
Not identified	70 (84.3)	61 (93.8)	9 (50.0)	Ref	63 (91.3)	7 (50.0)	Ref
**Desmoplasia, No. (%)**							
Present	5 (6.9)	3 (5.3)	2 (13.3)	2.47 (0.56–10.99)	3 (5.0)	2 (16.7)	2.35 (0.51–10.839)
Not identified	67 (93.1)	54 (94.7)	13 (86.7)	ref	57 (95.0)	10 (83.3)	Ref
**Adjacent melanocytic nevus, No. (%)**							
Present	6 (8.1)	3 (5.1)	3 (20.0)	1.65 (0.46–5.90)	3 (4.8)	3 (25.0)	1.94 (0.52–7.22)
Not identified	68 (91.9)	56 (94.9)	12 (80.0)	Ref	59 (95.2)	9 (75.0)	Ref

*ALM* acral lentiginous melanoma, *HR* hazard ratio, *CI* confidence interval.

*Statistically significant with 95% CIs not including 1.00.

**Table 4:** Five-year recurrence-free survival, distant metastasis-free survival, and melanoma-specific survival in ALM and non-ALM patients.

	Total, No	5-year Recurrence, No. (%)	5-year RFS (%) (95% CI)	Distant Metastasis in 5 years, No. (%)	5-year DMFS (%) (95% CI)	Death in 5 years, No. (%)	5-year MSS (%) (95% CI)
*ALM*							
**T category**							
1a	18 (14.5)	1 (5.6)	94.4 (83.9–100)	1 (5.6)	94.4 (83.9–100)	1 (5.6)	94.4 (83.9–100)
1b	10 (8.1)	2 (20.0)	80.0 (55.2–100)	2 (20.0)	80.0 (55.2–100)	2 (20.0)	80.0 (55.2–100)
2a	29 (23.4)	0 (0.0)	100 (100–100)	0 (0.0)	100 (100–100)	0 (0.0)	100 (100–100)
2b	8 (6.5)	3 (37.5)	62.5 (29.0–96.1)	3 (37.5)	62.5 (29.0–96.1)	0 (0.0)	100 (100–100)
3a	11 (8.9)	2 (18.2)	81.8 (59.0–100)	0 (0.0)	100 (100–100)	0 (0.0)	100 (100–100)
3b	18 (14.5)	6 (33.3)	66.7 (44.9–88.4)	4 (22.2)	77.8 (58.6–97.0)	5 (27.8)	72.2 (51.5–92.9)
4a	11 (8.9)	3 (27.3)	72.7 (46.4–99.1)	3 (27.3)	72.7 (46.4–99.1)	3 (27.3)	72.7 (46.4–99.1)
4b	19 (15.3)	12 (63.2)	36.8 (15.2–58.5)	11 (57.9)	42.1 (19.9–64.3)	9 (47.4)	52.6 (30.2–75.1)
**Clinical stage**							
1	56 (45.2)	3 (5.4)	94.6 (88.8–100)	3 (5.4)	94.6 (88.8–100)	3 (5.4)	94.6 (88.8–100)
2	68 (54.8)	26 (38.2)	61.8 (50.2–73.3)	21 (30.9)	69.1 (58.1–80.1)	17 (25.0)	75.0 (64.7–85.3)
*Non-ALM*							
**T category**							
1a	8 (15.1)	0 (0.0)	100 (100–100)	0 (0.0)	100 (100–100)	0 (0.0)	100 (100–100)
1b	7 (13.2)	0 (0.0)	100 (100–100)	0 (0.0)	100 (100–100)	0 (0.0)	100 (100–100)
2a	10 (18.9)	1 (10.0)	90.0 (71.4–100)	0 (0.0)	100 (100–100)	0 (0.0)	100 (100–100)
2b	2 (3.8)	0 (0.0)	100 (100–100)	0 (0.0)	100 (100–100)	0 (0.0)	100 (100–100)
3a	10 (18.9)	2 (20.0)	80.0 (55.2–100)	2 (20.0)	80.0 (55.2–100)	0 (0.0)	100 (100–100)
3b	3 (14.3)	2 (66.7)	33.3 (0.0–86.7)	2 (66.7)	33.3 (0.0–86.7)	2 (66.7)	33.3 (0.0–86.7)
4a	5 (9.4)	3 (60.0)	40.0 (0.0–82.9)	2 (40.0)	60.0 (17.1–100)	2 (40.0)	60.0 (17.1–100)
4b	8 (15.1)	5 (62.5)	37.5 (0.4–72.5)	3 (37.5)	62.5 (29.0–91.5)	2 (25.0)	75.0 (45.0–100)
**Clinical stage**							
1	25 (47.2)	1 (4.0)	96.0 (88.3–100)	0 (0.0)	100 (100–100)	0 (0.0)	100 (100–100)
2	28 (52.8)	12 (42.9)	57.1 (38.8–75.5)	9 (32.1)	67.9 (50.6–85.2)	6 (21.4)	78.6 (63.4–93.8)

*RFS* recurrence-free survival, *DMFS* distant metastasis-free survival, *MSS* melanoma-specific survival.

#### Risk factors of recurrence and distant metastasis in ALM

The prognostic value of clinicopathological variables in ALM and non-ALM patients were summarized in Tables 2 and 3, and Tables S1 and S2 in Supplementary Material. Notably, ALM patients having received SLNB experienced significantly less distant metastases in 5 years than its non-ALM counterpart. Microscopically, ALM lesions more than 4 mm thick, the presence of ulceration, and neurotropism, were related to higher incidences of recurrence and distant metastasis in 5 years. Sex, age, the implementation of SLNB, lymphovascular invasion, lymphocytic infiltration, tumor regression, desmoplasia, or adjacent nevi at presentation were unrelated to significant change in any endpoint in ALM patients.

#### Risk factors of recurrence and distant metastasis in non-ALM cutaneous melanoma

In non-ALM patients, diagnoses made at or older than 65 years of age had a higher rate of experiencing recurrences in 5 years. Regarding pathological features, ulcerations in non-ALM lesions implied higher rates of recurrence and distant metastasis in 5 years. Sex, location of primary lesions, thickness, lymphovascular invasion, lymphocytic infiltration, regression, desmoplasia, and adjacent nevi were unrelated to significant change in any endpoint in non-ALM patients.

Results of Cox univariate and multivariate analyses regarding prognostic factors for recurrence, distant metastasis, and MSS in 5 years among ALM and non-ALM patients, are shown in Table 5 and Supplementary Table S3, respectively. Among ALM patients, 6 factors were covered (tumor thickness, ulceration, mitotic rate, neurotropism, lymphovascular invasion, and the implementation of SLNB) in the multivariate analysis, among which the presence of ulceration remained significantly associated with higher recurrence and distant metastasis rates in 5 years, as well as a shorter MSS in 5 years. Tumor thickness of over 4 mm remained significantly linked to a higher distant metastasis rate in 5 years and a shorter MSS in 5 years.

**Table 5:** Univariate and multivariate analyses of prognostic factors for recurrence, distant metastasis, and melanoma-specific survival in 5 years among ALM patients.

Variables	Univariate HR (95% CI)	Univariate *p* value	Multivariate HR (95% CI)	Multivariate *p* value
**Recurrence in 5 years**				
Thickness > 4 mm	6.12 (1.77–21.18)*	0.0043*	0.44 (0.03–6.10)	0.5390
Ulceration	5.85 (2.58–13.23)*	< 0.0001*	11.72 (1.30–105.63)*	0.0283*
Mitosis ≥ 3/mm^2^	2.49 (0.92–6.76)	0.0729	1.49 (0.66–3.36)	0.3381
Neurotropism	14.08 (4.65–42.60)*	< 0.0001*	3.44 (0.69–17.11)	0.1315
**Distant metastasis in 5 years**				
SLNB performed	0.37 (0.15–0.94)*	0.0358*	0.76 (0.25–2.29)	0.6225
Thickness > 4 mm	5.46 (1.56–19.07)*	0.0078*	0.00 (0.00–0.00)*	< 0.0001*
Ulceration	6.45 (2.55–16.32)*	< 0.0001*	> 99.00*	< 0.0001*
Neurotropism	12.70 (3.81–42.34)*	< 0.0001*	7.19 (0.46–112.05)	0.159
Lymphovascular invasion	3.09 (0.88–10.88)*	0.0785*	0.89 (0.14–5.85)	0.9057
**Shorter melanoma-specific survival in 5 years**				
SLNB performed	0.32 (0.12–0.88)*	0.0276*	0.53 (0.12–2.28)	0.3944
Thickness > 4 mm	4.37 (1.23–15.54)*	0.0228*	0.00 (0.00–0.00)*	< 0.0001*
Ulceration	4.48 (1.72–11.69)*	0.0022*	> 99.00*	< 0.0001*
Neurotropism	12.40 (3.32–46.26)*	0.0002*	2.60 (0.70–9.67)	0.155

*ALM* acral lentiginous melanoma, *HR* hazard ratio, *CI* confidence interval, *SLNB* sentinel lymph node biopsy.

*Statistically significant.

With respect to non-ALM patients, 6 factors were covered (male gender, diagnoses made over 65 years of age, head and neck lesions, ulceration, neurotropism, and non-brisk lymphocytic infiltration) in the multivariate analysis. The presence of ulceration remained significantly associated with higher recurrence and distant metastasis rates in 5 years. Neurotropism remained significantly linked to a higher recurrence rate in 5 years. Additionally, male gender, head and neck lesions, ulceration, neurotropism, and non-brisk lymphocytic infiltration were significantly linked to shorter 5-year MSS in non-ALM patients.

## Discussion

Among the various subtypes of cutaneous melanoma, ALM stands out as a unique entity which features multiple distinctive characteristics, is often more advanced stage at diagnosis, and often has poorer prognosis^14,15^. The pathogenesis of ALM has not been fully clarified but is believed to differ from that of other melanoma subtypes. Ultraviolet (UV)-induced characteristics is less often found in ALM, implying the less significant effect of UV on the pathogenesis of ALM compared to other subtypes of cutaneous melanoma^16^. Previous Scottish, Japanese, Korean, and Taiwanese studies have identified mechanical stress as a unique precipitating factor of ALM, with more ALM lesions having been observed in physically stressed sites, such as the center of the heels and front of the foot^17–20^. Additionally, well-recognized risk factors of cutaneous melanoma, including a personal or family history of melanoma, fair skin, preexisting melanocytic nevi, are less significant in ALM^18,21^.

The poorer prognosis of ALM compared to other melanoma subtypes has been pointed out in previous cohorts. In Taiwan, the 5-year survival rate of ALM has been reported to be 39% in one study and 45.63% in another^1,3^. Thus, identifying prognostic factors early in the disease course is of great importance. The present cohort serves as one of the largest studies of ALM in literature, putting together clinical and pathological data of 125 ALM and 53 non-ALM melanoma patients. In the present study, the mean age of ALM at diagnosis was 63.7 years old, matching the number reported in a previous Chinese study, but older than that in a Korean study and younger than in a Caucasian study^5,17,18^. A modest female predominance was observed, with a male-to-female ratio of 1:1.33, which was similar to previous Caucasian reports, but differed from that in Chinese patients^5,7,17^. Compared with those in Korean and Chinese patients, ALM lesions in Taiwanese patients in this study had a similar rate of ulceration (49.6% vs. 42.3% and 47.9%) and a slightly lower proportion of lesions thicker than 4 mm (32.0% vs. 32.9% and 40.8%)^5,18^. The mean Breslow thickness in ALM lesions in this study was 3.17 mm, thinner than that reported in the aforementioned Chinese study yet thicker than cutaneous melanoma in general, which reflected the late timing of diagnosis in ALM^5^.

The prognostic value of several clinical and pathological characteristics in ALM and non-ALM patients was identified in this study. Regarding the practice of SLNB in this study, SLNB was performed in 84.7% of ALM patients, aiding the establishment of treatment plans for this grave condition. Apart from 15 non-ALM patients with non-ulcerated melanoma lesions 0.8–1 mm thick, or with ulcerated lesions less than 0.8 mm thick at diagnosis, 38 non-ALM patients could benefit from SLNB. SLNB was performed in 76% of these 38 patients. A recent Taiwanese study revealed that SLNB was linked to favorable outcomes in clinically node-negative cutaneous melanoma, particularly in ALM^22^. In that study, 78.9% of ALM patients and 57.8% of non-ALM patients received SLNB. In comparison, the percentage of patients having received SLNB was higher in the present study. SLNB results were intrinsically negative in all 124 ALM patients and 53 non-ALM patients, corresponding with the clinical stages, 1 and 2, at diagnosis of these patients. The inclination of lower distant metastasis rate in 5 years and longer MSS in 5 years in ALM patients having received SLNB might have resulted from early clearance of undetected melanoma or more vigilant surveillance during follow-up. However, a larger sample size in future studies could help clarify the prognostic value of the implementation of SLNB due to its insignificance in multivariate analyses.

Thickness of more than 4 mm was associated with poorer outcome in ALM patients in this study. While thickness was linked to worse prognosis in some cohorts, its significance remained equivocal and eclipsed by that of SLNB^7,23,24^. Thicker skin in acral regions also impaired the accuracy of thickness measurement of ALM lesions^25^. On the other hand, adverse prognostic features in non-ALM cutaneous melanoma observed in this study include male gender, head and neck lesions, ulceration, neurotropism, and non-brisk lymphocytic infiltration, which add to the comprehensiveness of current endeavor to pinpoint unfavorable features in cutaneous melanoma^26–29^. However, a larger sample size could elucidate the prognostic power of these variables.

DMFS and MSS of around 80% were observed in ALM patients. Several efficacious systemic therapies were developed in the past 2 decades; however, there were a few factors that prevented longer survival in ALM patients with distant metastasis. Mutation frequencies of reported melanoma driver genes varied remarkably among different melanoma subtypes. *BRAF* mutations were most commonly found in non-ALM, while *KIT* mutations were more prevalent in ALM, which helped to explain why ALM responded poorly to *BRAF* inhibitors^30^. Moreover, a lower mutation burden was found in ALM. Co-occurrence of several structural variants was identified in ALM, which changed the phenotype of cancer cells, making ALM more resistant to therapy. The incidence of cytoband gains was significantly higher in ALM than in non-ALM, which also contributed to the poorer prognosis in ALM^31^. On the other hand, pembrolizumab and nivolumab were covered by National Health Insurance (NHI) in Taiwan since 2000, while vemurafenib was covered since 2015, and dabrafenib and trametinib were included in NHI coverage starting from 2021. With novel FDA-approved medication included in NHI coverage in Taiwan only recently, patients receiving them were only gradually accumulating in number. The efficacy of these novel agents could hopefully be evident in cohorts in the near future.

In sum, this study correlated clinicopathological characteristics and the prognosis of cutaneous melanoma, with a focus on ALM. Further clinical and pathological studies across Taiwan and the world could help solidify and optimize current understanding on the prognostic factors of ALM and other subtypes of cutaneous melanoma. Molecular studies are anticipated to unveil the mechanisms underlying these risk factors.

### Strengths and limitations

Strengths of this study were its well-dispersed patient distribution across Taiwan, reflecting the demographics of primary melanoma population in Taiwan. Our study had a few limitations. The single-center design limited the generalizability of this study. Retrospectively collected data were subject to misclassification bias. A meticulous review of medical record and pathological reports had been conducted to circumvent these pitfalls, but a larger sample size could help assess the prognostic significance of each clinical or pathological characteristic more comprehensively.

## Materials and methods

### Patient selection and data collection

This is a retrospective cohort approved by the Research Ethics Committee of National Taiwan University Hospital (NTUH-REC) (Approval serial number: 202008059RINB). This study conforms to the ethical norms and standards in the Declaration of Helsinki. A waiver of informed consent was given by NTUH-REC. From the electronic medical record database, 1238 entries with diagnoses of cutaneous melanomas between January 1, 2004, and December 31, 2020 at National Taiwan University Hospital were obtained using a computer-assisted search. Entries documenting multiple appointments of the same patient were excluded (n = 850), keeping merely information of the latest encounter. Cases without a formal pathology report or lacking description on the Breslow thickness of melanoma lesions were excluded (n = 227). Patients under 20 years old at diagnosis and those diagnosed before 2004, since when SLNB has been universally conducted in patients with appropriate indications in NTUH, were excluded. Patients initially staged melanoma in situ, stage 3, and stage 4 in the 8th AJCC staging system were excluded. After excluding the cases above, electronic medical records including formal pathological reports of 177 consecutive patients remained.

Patients included in this study received standard-of-care treatment according to the latest National Comprehensive Cancer Network (NCCN) Clinical Practice Guidelines in Oncology. Clinical data regarding patient demographics, clinical descriptions of the lesion, including the size, characteristics, and location, the 8th AJCC T category and stage, the clinical course recorded at follow-up clinics were collected through electronic medical records and the Cancer Registry of the Medical Information Management Office of National Taiwan University Hospital.

RFS denoted the period during which no local recurrence, regional recurrence, distant metastasis, or death was reported^32^. DMFS represented the length of follow-up during which no distant metastases were discovered. MSS represented the duration from diagnosis to the time when death due to melanoma was recorded. Thickness of lesions was stratified as ≤ 1 mm, 1–2 mm, 2–4 mm, and > 4 mm. Mitotic rate was categorized as follows: < 1 mitosis/mm^2^, 1–3 mitosis/mm^2^, and ≥ 3 mitosis/mm^2^. Lymphocytic infiltration was stratified as none, non-brisk lymphocytic infiltration, and brisk lymphocytic infiltration.

### Statistical analysis

Associations between variables of interest and clinical endpoints were analyzed with the Fisher exact test or Chi-square test when indicated. The impact of variables on endpoints was assessed using univariate and multivariate Cox proportional hazard models. In the multivariate analysis, inclusion of variables with univariate P values of less than 0.1, and exclusion of variables with excessive missing data, were implemented. All tests were two-sided. P values of less than 0.05 were regarded as statistically significant. Analyses were performed using SAS 9.4 (Cary, North Carolina, USA).

## Supplementary Information

Supplementary Information.
