# Supplementary material for: Risk factors of recurrence and distant metastasis in primary cutaneous melanoma in Taiwan
Source: Sci Rep. 2021 Oct 25;11:21012. doi: 10.1038/s41598-021-00386-4 (PMC8545938; doi:10.1038/s41598-021-00386-4)
Supplement: Supplementary file 1 — Supplementary Information. [file 41598_2021_386_MOESM1_ESM.pdf]

Supplementary Material

Table S1. Clinical risk factors of recurrence and distant metastases in 5 years among non-ALM patients

Variables	Total	No recurrence	Recurrence in 5 years	HR (95% CI)	No metastasis	Distant Metastasis in 5 years	HR (95% CI)
Total, No. (%)	53 (100)	40 (75.5)	13 (24.5)		44 (83.0)	9 (17.0)	
Sex, No. (%)							
Male	24 (45.3)	15 (37.5)	9 (69.2)	3.05 (0.94-9.92)	18 (40.9)	6 (66.7)	2.53 (0.63-10.14)
Female	29 (54.7)	25 (62.5)	4 (30.8)	ref	26 (59.1)	3 (33.3)	ref
Age, No (%), y							
<65	37 (69.8)	31 (77.5)	6 (46.2)	ref	32 (72.7)	5 (55.6)	ref
≧65	16 (30.2)	9 (22.5)	7 (53.8)	* 4.41 (1.46-13.29)	12 (27.3)	4 (44.4)	2.72 (0.73-10.24)
Location, No. (%)							
Trunk	19 (35.9)	14 (35.0)	5 (38.5)	ref	16 (36.4)	3 (33.3)	ref
Head and neck	9 (17.0)	4 (10.0)	5 (38.5)	2.60 (0.74-9.13)	5 (11.4)	4 (44.4)	3.96 (0.86-18.19)
Extremities	25 (47.2)	22 (55.0)	3 (23.1)	0.33 (0.08-1.39)	23 (52.3)	2 (22.2)	0.40 (0.07-2.43)
Acral	0 (0.0)	0 (0.0)	0 (0.0)	NA	0 (0.0)	0 (0.0)	NA
SLNB, No. (%)							
Performed	29 (55.8)	23 (59.0)	6 (46.2)	0.60 (0.20-1.80)	24 (55.8)	5 (55.6)	0.91 (0.24-3.39)
Not performed	23 (44.2)	16 (41.0)	7 (52.8)	ref	19 (44.2)	4 (44.4)	ref

ALM: acral lentiginous melanoma. SLNB: sentinel lymph node biopsy. HR: hazard ratio. CI: confidence interval. * Statistically significant.

Table S2. Pathological risk factors of recurrence and distant metastasis in 5 years among non-ALM patients

	Total	No recurrence	Recurrence in 5 years	HR (95% CI)	No metastasis	Distant Metastasis in 5 years	HR (95% CI)
Total, No. (%)	53 (100)	40 (75.5)	13 (24.5)		44 (83.0)	9 (17.0)	
Thickness, No. (%), mm							
<=1	15 (28.3)	15 (37.5)	0 (0.0)	ref	15 (34.1)	0 (0.0)	ref
>1, <=2	13 (24.5)	12 (30.0)	1 (7.7)	NA	13 (29.6)	0 (0.0)	1
>2, <=4	15 (28.3)	10 (25.0)	5 (38.5)	NA	11 (25.0)	4 (44.4)	NA
>4	10 (18.9)	3 (7.5)	7 (53.8)	NA	5 (11.4)	5 (55.6)	NA
Ulceration, No. (%)							
Present	14 (26.4)	7 (17.5)	7 (53.8)	* 7.15 (2.37-21.58)	9 (20.5)	5 (55.6)	* 7.46 (1.98-28.18)
Absent	39 (73.6)	33 (82.5)	6 (46.2)	ref	35 (79.5)	4 (44.4)	ref
Mitosis, No. (%), /mm2							

<1	10 (27.0)	9 (32.1)	1 (11.1)	ref	9 (29.3)	1 (16.7)	ref
>=1,<3	0 (0.0)	0 (0.0)	0 (0.0)	NA	0 (0.0)	0 (0.0)	NA
>=3	27 (73.0)	19 (67.9)	8 (88.9)	4.43 (0.55-35.5)	22 (71.0)	5 (83.3)	2.63 (0.31-22.54)
Lymphovascular invasion, No. (%)							
present	1 (3.7)	1 (4.8)	0 (0.0)	NA	1 (4.4)	0 (0.0)	NA
not identified	26 (96.3)	20 (95.2)	6 (100)	ref	22 (95.6)	4 (100)	ref
Lymphocytic infiltration, No. (%)							
none	1 (3.3)	0 (0.0)	1 (12.5)	ref	0 (0.0)	1 (20.0)	ref
non-brisk	26 (86.7)	19 (86.4)	7 (87.5)	0.35 (0.04-3.00)	22 (88.0)	4 (80.0)	0.20 (0.02-1.93)
brisk	3 (10.0)	3 (13.6)	0 (0.0)	0	3 (12.0)	0 (0.0)	0
Regression, No. (%)							
present	7 (25.9)	6 (28.6)	1 (16.7)	0.49 (0.06-4.21)	7 (30.4)	0 (0.0)	0
not identified	20 (74.1)	15 (71.4)	5 (83.3)	ref	16 (69.6)	4 (100)	ref
Neurotropism, No. (%)							
present	3 (11.1)	1 (4.8)	2 (33.3)	5.29 (0.96-29.22)	2 (8.7)	1 (25.0)	3.85 (0.40-37.45)
not identified	24 (88.9)	20 (95.2)	4 (66.7)	ref	21 (91.3)	3 (75.0)	ref
Desmoplasia, No. (%)							
present	1 (3.7)	0 (0.0)	1 (16.7)	2.71 (0.32-23.37)	1 (4.4)	0 (0.0)	0
not identified	26 (96.3)	21 (100)	5 (83.3)	ref	22 (95.6)	4 (100)	ref
Adjacent melanocytic nevus, No. (%)							
present	12 (40.0)	11 (47.8)	1 (14.3)	0.17 (0.02-1.43)	11 (44.0)	1 (20.0)	0.26 (0.03-2.35)
not identified	18 (60.0)	12 (52.2)	6 (85.7)	ref	14 (56.0)	4 (80.0)	ref

ALM: acral lentiginous melanoma. HR: hazard ratio. CI: confidence interval. * Statistically significant.

Table S3. Univariate and multivariate analyses of prognostic factors for recurrence, distant metastasis, and melanoma-specific survival in 5 years among non-ALM patients

**Recurrence in 5 years**				
Variables	Univariate HR (95% CI)	Univariate P-value	Multivariate HR (95% CI)	Multivariate P-value
Male gender	3.05 (0.94-9.92)	0.064	1.51 (0.31-7.28)	0.6091
Age≧65	* 4.41 (1.46-13.29)	0.0085	1.01 (0.99-1.03)	0.4108
Ulceration	* 7.15 (2.37-21.58)	0.0005	* 15.45 (1.78-134.30)	* 0.0131
Neurotropism	5.29 (0.96-29.22)	0.0559	* 3.32 (1.18-9.33)	* 0.0231
**Distant metastasis in 5 years**				
Variables	Univariate HR (95% CI)	Univariate P-value	Multivariate HR (95% CI)	Multivariate P-value
Head and neck lesions	3.96 (0.86-18.19)	0.077	1.87 (0.28-12.28)	0.5152
Ulceration present	* 7.46 (1.98-28.18)	* 0.003	* 5.22 (1.06-25.87)	* 0.0428
**Shorter 5-year melanoma-specific survival**				

Variables	Univariate HR (95% CI)	Univariate P-value	Multivariate HR (95% CI)	Multivariate P-value
Male gender	6.25 (0.73-53.58)	0.0945	* 0.00 (0.00-0.00)	* <0.0001
Head and neck lesions	8.85 (0.98-80.02)	0.0522	* > 99.00	* <0.0001
Ulceration	* 11.02 (2.00-60.88)	* 0.0059	* > 99.00	* <0.0001
Non-brisk lymphocytic infiltration	0.07 (0.00-1.04)	0.0533	* 0.10 (0.02-0.55)	* 0.008
Neurotropism	10.58 (0.64-173.86)	0.0985	* > 99.00	* <0.0001

ALM: acral lentiginous melanoma. HR: hazard ratio. CI: confidence interval. * Statistically significant.
